# MID-LIFE PLASMA PROTEINS ASSOCIATE WITH PREFRAILTY AND FRAILTY: A PROTEOMIC ANALYSIS

**DOI:** 10.1093/geroni/igad104.2243

**Published:** 2023-12-21

**Authors:** Fangyu Liu, Jennifer Schrack, Jeremy Walston, Rasika Mathias, Josef Coresh, Keenan Walker

**Affiliations:** Johns Hopkins Bloomberg School of Public Health, Baltimore, Maryland, United States; Johns Hopkins University, Baltimore, Maryland, United States; Johns Hopkins University, Baltimore, Maryland, United States; Johns Hopkins University, Baltimore, Maryland, United States; Johns Hopkins University, Baltimore, Maryland, United States; National Institute on Aging, National Institutes of Health, Baltimore, Maryland, United States

## Abstract

Physical frailty is a syndrome that typically manifests in later life, although the pathogenic process causing physical frailty likely begins decades earlier. To date, few studies have examined the biological signatures in mid-life associated with frailty in late life. We evaluated 4955 plasma proteins (log 2-transformed and standardized) measured using the SomaScan platform among 3810 participants (57.8+/-5.0 years, 58.2% women) at Visit 3 of the Atherosclerosis Risk in Community (ARIC) study, and their frailty status across up to 3 study visits in late-life (median follow-up 19 years). We used multinomial logistic regression models to examine the associations between the protein levels and the frailty status, adjusting for demographics, health indicators, comorbidities, and follow-up time. Two sets of 2 proteins were identified to be associated with prefrailty and frailty, respectively, after Bonferroni correction (p< 1.01×10-5). Higher concentration of hydroxymethylglutaryl-CoA synthase (HMGCS1; OR=1.22), a regulator of cholesterol synthesis, and lower concentration of dual specificity protein phosphatase 13 isoform A (DUSP13A; OR=0.82), a protein involved in cell apoptosis, were associated with higher odds of prefrailty. Higher concentration of growth differentiation factor 15 (GDF15; OR=1.39), a protein related to inflammation and cell senescence, and lower concentration of contactin-1 (CNTN1; OR=0.72), a protein involved in nervous system development and signalling, were associated with higher odds of frailty. The pathway and upstream regulator analyses of the physical frailty- or prefrailty-associated proteins (p< 0.01) revealed the dysregulation of apoptosis, inflammation, and neuromuscular communication in mid-life. Our findings provides new insights into frailty etiology earlier in the life course.

